# Rapid Measurement of Antioxidant Properties of *Dendrobium officinale* Using Near-Infrared Spectroscopy and Chemometrics

**DOI:** 10.3390/foods13111769

**Published:** 2024-06-05

**Authors:** Xiaoqing Cao, Jing Huang, Jinjing Chen, Ying Niu, Sisi Wei, Haibin Tong, Mingjiang Wu, Yue Yang

**Affiliations:** Zhejiang Provincial Key Laboratory for Water Environment and Marine Biological Resources Protection, College of Life and Environmental Science, Wenzhou University, Wenzhou 325035, China; caoxiaoqing0311@163.com (X.C.); jinghuang0224@163.com (J.H.); 15381561582@163.com (J.C.); naive916@163.com (Y.N.); weisisi1511@163.com (S.W.); hbtong@wzu.edu.cn (H.T.); wmj@zeu.edu.cn (M.W.)

**Keywords:** *Dendrobium officinale*, antioxidant activity, near-infrared spectroscopy, chemometrics

## Abstract

*Dendrobium officinale* (*D. officinale*), often used as a dual-use plant with herbal medicine and food applications, has attracted considerable attention for health-benefiting components and wide economic value. The antioxidant ability of *D. officinale* is of great significance to ensure its health care value and safeguard consumers’ interests. However, the common analytical methods for evaluating the antioxidant ability of *D. officinale* are time-consuming, laborious, and costly. In this study, near-infrared (NIR) spectroscopy and chemometrics were employed to establish a rapid and accurate method for the determination of 2,2′-azinobis-3-ethylbenzothiazoline-6-sulfonic acid (ABTS) scavenging capacity, 2,2-diphenyl-1-picrylhydrazyl (DPPH) scavenging capacity, and ferric reducing antioxidant power (FRAP) in *D. officinale*. The quantitative models were developed based on the partial least squares (PLS) algorithm. Two wavelength selection methods, namely the genetic algorithm (GA) and competitive adaptive reweighted sampling (CARS) method, were used for model optimization. The CARS-PLS models exhibited superior predictive performance compared to other PLS models. The root mean square errors of cross-validation (*RMSECVs*) for ABTS, FRAP, and DPPH were 0.44%, 2.64 μmol/L, and 2.06%, respectively. The results demonstrated the potential application of NIR spectroscopy combined with the CARS-PLS model for the rapid prediction of antioxidant activity in *D. officinale*. This method can serve as an alternative to conventional analytical methods for efficiently quantifying the antioxidant properties in *D. officinale*.

## 1. Introduction

*Dendrobium officinale* (*D. officinale*), belonging to the *Dendrobium* genus of the Orchidaceae family, has long been used as a healthy tea, beverage, or cooking spice. It is mainly cultivated in Zhejiang, Anhui, and Yunnan provinces in China and differs in quality and prices with different origins. *D. officinale*, known as “the first of the nine Chinese fairy herbs” [1,2,3], is a rich source of polysaccharides, polyphenols, total flavonoids, and alkaloids which are associated with antioxidant, antitumor, and hypoglycemic pharmacological activities. Recently, several new components have also been isolated and identified from *D. officinale*, such as chromcinale and leonuriside C [4]. Among the multiple components, the polysaccharide content accounts for 35% in *D. officinale*, which can scavenge free radicals of the body and has strong antioxidant activity [5,6]. The demand for high-quality *D. officinale* has increased rapidly with the attention to nutritional value, and now, it is used in a variety of supplementary foods and functional drinks.

*D. officinale*’s antioxidant activity is an important issue for consumers and is associated with the contents of polysaccharides and polyphenols in *D. officinale*, which are influenced by geographical origins and harvesting times [1,2]. Therefore, it is imperative to determine the antioxidant activity capacity of *D. officinale* to safeguard consumers’ interests. However, the common analytical methods for bioactive substance are always complicated, time-consuming, and laborious, waste reagents, and pollute the environment, mainly including ultraviolet–visible spectrophotometry (UV-Vis) and high-performance liquid chromatography (HPLC) [7,8,9,10]. Therefore, it is necessary to develop a more efficient, environmentally friendly, and convenient method to assess the antioxidant activity of *D. officinale*.

Since the late 1980s, near-infrared (NIR) spectroscopy has prevailed over traditional analytical methods with many advantages and has become a good alternative. For example, it involves easily operated instruments, simple sample pre-processing, and fast detection speeds. The absorption spectrum of NIR lies in range of 1000–2500 nm and is related to the hydrogen-containing functional groups such as C-H, N-H, O-H, and S-H [11,12,13]. Due to the fact that bioactive components contain large amounts of the above-mentioned hydrogen-containing groups, NIR information can be associated with these chemical parameters to develop a quantitative analytical model. At present, NIR spectroscopy technology is widely used in agriculture [14,15,16], food [11,17,18,19], medicine [20,21,22], petrochemistry [23,24,25], and other fields. There are numerous studies confirming that NIR has good potential to successfully predict the antioxidant activity of Chinese medicine. For example, Yi et al. used NIR spectroscopy to quantify the 2,2′-azion-bis-3-ethylbenzothiazoline-6-sulfonic acid (ABTS), ferric reducing antioxidant power (FRAP), and 1,1-diphenyl-2-picrylhydrazyl (DPPH) antioxidant activities of *Poria cocos* at the same time, and achieved a good prediction effect [21]. Yang et al., using NIR spectroscopy, successfully predicted polysaccharides, polyphenols, total flavonoids, and total alkaloids in *D. officinale* [26]. However, to our knowledge, few studies have applied NIR technology to quantify the antioxidant activity of *D. officinale.*

The NIR spectrum features serious overlapping peaks and weak absorption. Thus, chemometrics should be applied to separate and extract key spectrum data information and to remove the spectral baseline drift caused by the sample particle size [1]. Apart from useful spectrum data, the full NIR spectrum may contain many irrelevant variables, which reduce the robustness and prediction accuracy of calibration models [27,28,29,30]. Genetic algorithms (GAs) and competitive adaptive reweighted sampling (CARS) have obtained wide acceptance among variable selection algorithms [1,20,27]. Several studies have confirmed that GAs have obtained good results in the screening of characteristic variables [31]. CARS, a fast calculation method proposed by Li et al., is a powerful tool for dealing with complex analytical systems [32].

In this study, a combination of NIR spectroscopy and chemometrics was employed to simultaneously predict the antioxidant activity of ABTS, FRAP, and DPPH in *D. officinale*. The specific objectives are to (1) measure the antioxidant activities of ABTS, FRAP, and DPPH in *D. officinale*; (2) compare the performance of different pretreatment methods on the NIR spectrum; and (3) investigate the optimization ability of GA and CARS in developing quantitative models for the antioxidant activities of ABTS, FRAP, and DPPH in *D. officinale*.

## 2. Materials and Methods

### 2.1. Samples and Reagents

A total of 111 *D. officinale* samples were purchased online from various regions in China, including Yunnan, Anhui, and Zhejiang provinces. Each sample was washed with running water to remove surface impurities and dirt, followed by being dried in a constant-temperature drum wind dryer at 80 °C for 4.5 h. Then, the dried samples were crushed and passed through an 80-mesh sieve to obtain powder of *D. officinale* with particle sizes less than 180 μm. Finally, the powdered samples were placed in a sealed bag and stored in an atmospheric dryer (inner diameter: 210 mm; Huaou Glass Co., Ltd. (Yancheng, Jiangsu, China)) at the temperature of 25 °C for further analysis.

DPPH (96% purity), ABTS (98% purity), TPTZ (99% purity), ferrous sulfate heptahydrate (FeSO_4_), sodium acetate anhydrous, and potassium persulfate (K_2_S_2_O_8_) were obtained from Macklin Biochemical Technology Co., Ltd. (Shanghai, China). Ferric chloride (FeCl_3_) hexahydrate and absolute ethanol were provided from Xilong Science Co., Ltd. (Shantou, Guangdong, China). Glacial acetic acid and concentrated hydrochloric acid (HCl) were purchased from Zhongxing Chemical Reagent Co., Ltd. (Lanxi, Zhejiang, China). Ultrapure water was purified through a Milli-Q system (Millipore, MA, USA).

### 2.2. NIR Spectral Acquisition

The NIR spectra were collected in diffuse reflectance mode using a Fourier transform NIR spectrometer (Antaris II, Thermo Fisher Scientific, USA). About 2 g of the powdered sample was uniformly placed in the rotating quartz cup. Spectra were acquired in the range of 1000–2500 nm and the spectral resolution was 8 cm^−1^. The number of scans was 64 with air as the background. Each sample was collected 3 times, and the average spectra were obtained for further analysis.

### 2.3. Reference Assays

#### 2.3.1. Dendrobium Officinale Extraction

The *D. officinale* powder was accurately weighed to 50 mg and placed in a 50 mL beaker containing a constant volume of ultrapure water. Subsequently, the mixture was sonicated at room temperature for 20 min. Following this, all samples were centrifuge 3000 rpm for 10 min using a high-speed freezing centrifuge (5901R, Eppendirf, Germany), and the resulting supernatants were considered as *D. officinale* extracts, which were then stored in the refrigerator at 4 °C.

#### 2.3.2. ABTS Test

With reference to the method given by Muhammad et al. [33], in this assay, an acetic acid buffer of pH 4.5 was used to prepare the ABTS solution and K_2_S_2_O_8_ solution, which could be mixed proportionally to make an ABTS reaction solution for determination. In addition, in order to calibrate the UV-Vis spectrophotometer, a standard solution of potassium dichromate in a concentration range of 20–100 mg kg^−1^ was utilized. To be specific, 100 mL of the ABTS solution (7 mM) was mixed with 1.76 mL of the K_2_S_2_O_8_ solution (140 mM) and a dark reaction was performed at room temperature for 12 h. The mixture was then diluted with ultrapure water until the absorbance at 734 nm was 0.70 ± 0.02 to obtain an ABTS working liquid. Finally, 4 mL of the ABTS working liquid was mixed with 200 µL of the sample solution and left for 30 min in the dark at 25 °C. With ultrapure water as the blank, the absorbance of samples was measured at 734 nm with a UV-Vis spectrophotometer (UV-1810, Puxi, China). Each sample was measured three times and the clearance capacity (%) was calculated by following formula:

Clearance capacity (%) = [A_0_ − (A_1_ − A_2_)]/A_0_ × 100

where A_0_ is the absorbance of the blank group, A_1_ is the absorbance of the sample solution mixed with the ABTS working liquid, and A_2_ is the absorbance of the sample solution without the ABTS working liquid.

#### 2.3.3. FRAP Test

The FRAP reagent, following the method of Frankel et al. [34], consisted of a mixture of acetate buffer (0.3 mol/L), TPTZ (10 mM in 40 mM HCl), and FeCl_3_ (20 mM) at a ratio of 10:1:1. Subsequently, 3.6 mL of the FRAP reagent was combined with 400 μL of the sample solution, followed by standing at 37 °C for 30 min. The absorbance at 593 nm was then measured using a UV-Vis spectrophotometer to determine the sample’s clearance capacity of ferric ion. All samples were measured three times in parallel to obtain the mean value. The clearance capacity was denoted by the FeSO_4_ concentration (μM). The linear regression formula for FRAP is y = 0.0019x + 0.0044, *r^2^* = 0.9995, with a range of 50–500 μM.

#### 2.3.4. DPPH Test

Referring to the experimental method of Guo et al. [35], 2 mL of the sample solution was mixed with 2 mL of DPPH ethanol solution (0.1 mM), shaken well, and reacted for 30 min at room temperature in the absence of light. The absorbance of the sample was measured at 517 nm using a UV-Vis spectrophotometer, with ultrapure water used as a blank. Each sample was measured three times, and the DPPH clearance capacity (%) was calculated as follows:

Clearance capacity (%) = [A_0_ − (A_1_ − A_2_)]/A_0_ × 100

where A_0_ represents the absorbance of the DPPH ethanol solution without any sample solution, A_1_ represents the absorbance of the sample solution containing the DPPH ethanol solution, and A_2_ represents the absorbance of the sample solution containing ethanol solution.

### 2.4. Spectral Pretreatment Methods

Due to physical variations, irrelevant information such as background noise, the dark current of the instrument, and particle size and shape can interfere with the raw spectrum [36,37]. Therefore, it is crucial to use spectral pre-processing methods to remove irrelevant information and noise. In this study, several potential spectral processing methods were utilized to deal with these impacts on the model. The first derivative (1D) can reduce translation signals independently of the wavelength [38]. However, using derivative methods alone may introduce noise and reduce the signal-to-noise ratio; thus, they are combined with Savitsky-Golay (SG) smoothing [39]. Additionally, multiplicative scatter correction (MSC) separates scattered signals from the absorbed signal by utilizing the same scattering coefficient in order to eliminate the spectral scattering effect and slope change [40]. The standard normal variate (SNV) standardizes various variables in the NIR diffuse reflection to eliminate the impact of scattering and sample particle variation [41].

### 2.5. Wavelength Selection Methods

#### 2.5.1. GA

The GA, a promising method for wavelength selection, operates based on the principle of the survival of the fittest in nature [42]. The implementation steps of the GA are as follows: parameter encoding; group initializing; design fitness function; design genetic manipulation; convergence criterion; and wavelength selection. The concept behind the GA wavelength selection method is to determine the fitness function by evaluating the model’s predictive ability using interactive validation methods. The implementation approach involves establishing a partial least squares (PLS) regression model with selected wavelength variables and selecting key variables through continuous genetic iteration using GA’s selection, exchange, and mutation operators while eliminating irrelevant or nonlinear variables. By simplifying the calibration model without compromising accuracy, its predictive ability and robustness can be improved.

#### 2.5.2. CARS Algorithm

The CARS algorithm is a feature variable selection method that combines the Monte Carlo sampling method with PLS model regression coefficients [32]. This algorithm is suitable for high-dimensional spectra as it gradually evaluates, analyzes, filters, and eliminates each wavelength point in the spectra. The implementation steps of CARS are as follows: (1) 80% of random samples are used as a calibration set for the PLS regression model; (2) wavelengths with smaller regression coefficients are removed using the exponentially decreasing function (EDF); (3) wavelengths with larger regression coefficients are screened out by adaptive reweighted sampling (ARS). Finally, the subset of wavelengths with the smallest root mean square error of cross-validation (*RMSECV*) values in the PLS model is selected.

### 2.6. Model Performance Evaluation

The prediction performance of the calibration model was evaluated using the following parameters: the correlation coefficient of the calibration set (*R*^2^*_C_*), the correlation coefficient of the prediction set (*R*^2^*_P_*), the root mean square error of the calibration set (*RMSEC*), and the root mean square error of the prediction set (*RMSEP*). In addition to the aforementioned assessment indicators, research has demonstrated the potential of other rigorous validation protocols for assessing model performance, such as leave-one-out cross-validation (Q2), stability assessment through bootstrapping experiments, regression line slope, and chance correlation [43,44,45]. Therefore, *R*^2^*_C_*, *R*^2^*_P_*, *RMSEC*, *RMSEP*, *RMSECV*, *Slope* value, *r*^2^*_m_* value, and y-randomization tests were utilized in this study to evaluate model performance. Among these metrics, the *r*^2^*_m_* value was used to exhibit the correlation between measured and predicted values. To better indicate the predictive performance of the calibration model, an *r*^2^*_m_* value with a threshold of 0.5 was computed using the following formula [44,46]:

$${r^{2}}_{m}=r^{2}\times (1-\sqrt{r^{2}-{r^{2}}_{0}})$$

where *r*^2^ and *r*^2^_0_ are the correlation coefficients between measured and predicted values, with and without the intercept, respectively.

The y-randomization test was employed to assess model robustness, and the *cR*^2^*_P_* parameter was calculated to quantify the difference between the y-randomization *R*^2^ (*R*^2^*_rand_*) and the original calibration model *R*^2^ (*R*^2^*_C_*). The threshold value of *cR*^2^*_P_* is 0.5, and its formula is as follows [45,46]:

(1) $$c{R^{2}}_{p}=R_{c}\sqrt{{R^{2}}_{c}-{R^{2}}_{rand}}$$

In general, higher values and closer proximity to 1 for *R*^2^*_C_* and *R*^2^*_P_* indicate a more precise fit. Additionally, lower *RMSEC*/*RMSEP* values suggest better predictive accuracy, thus indicating improved prediction ability and robustness for the NIR model.

## 3. Results and Discussion

### 3.1. NIR Spectral Features

**Figure 1:**
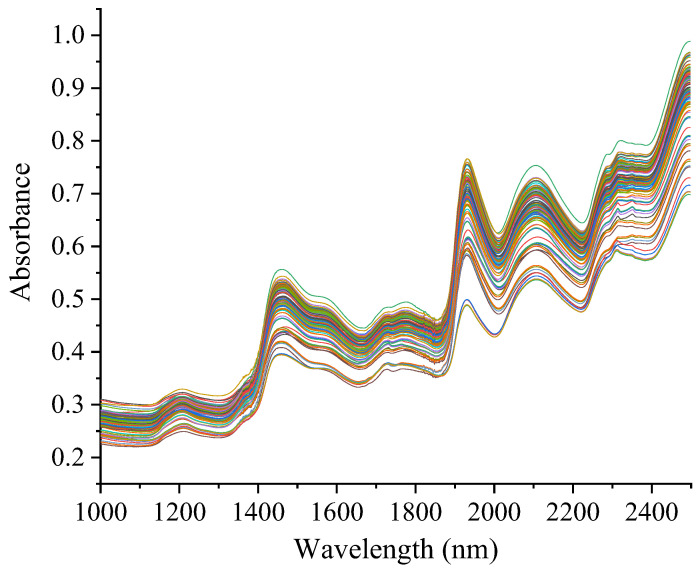
Raw near-infrared spectra of all *Dendrobium officinale* samples. Each line represents the near-infrared spectrum of each sample.

The raw NIR spectra of 111 *D. officinale* samples from different regions within 1000–2500 nm are shown in Figure 1. The intensive spectral peaks located at 1440 nm and 1940 nm corresponded to the deformation and stretching vibration of O-H groups [47]. Other intense absorption peaks were mainly observed around 1210 nm, 1730 nm, 2280 nm, and 2330 nm. The peaks around 1210 and 1730 nm were caused by the second overtone of C-H stretching vibration and the first overtone of the C-H stretching vibration [11], respectively. In addition, the broad peaks located at 2280 nm and 2330 nm were derived from a combination of C-H and -CH_2_ stretching and deformation vibrations.

### 3.2. Outlier Detection and Sample Partition

**Figure 2:**
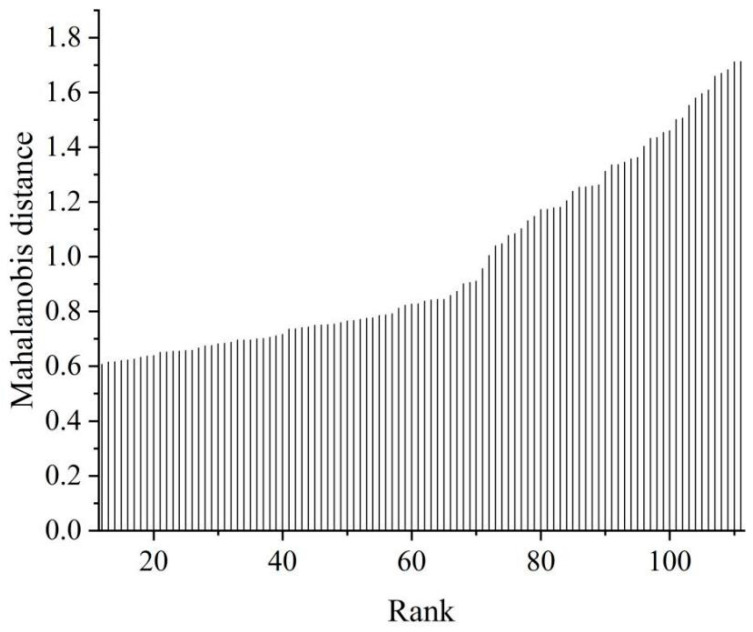
Plots of spectral outlier detection of ABTS.

It is difficult to identify spectral error by just visually examining the raw spectra, so an outlier detection method is necessary to reduce severe errors in the calibration model. In this study, the Mahalanobis distance was applied to discriminate outliers in the spectral data before NIR model building. The core idea of this method is to measure the distance between each sample spectrum and the average spectrum of all samples, and the Chauvenet test with a 95% confidence level is used to identify whether the sample spectrum is abnormal [48]. Finally, no outliers were found for ABTS, FRAP, and DPPH in *D. officinale*. Therefore, all samples were used to construct the calibration model for further analysis. In the case of ABTS, for example, the spectral error is shown in Figure 2, where it can be seen that all spectral data were within the thresholds.

To ensure an objective evaluation of the model performance, it is necessary to divide the samples into a calibration set and a prediction set. The calibration set is used to construct the model, while the prediction set is used to validate it. In this study, the Kennard and Stone (KS) method was employed to select calibration and prediction samples. The method operates by iteratively selecting samples with the greatest Euclidean distances from each other to form the calibration set until reaching the desired number of samples. The remaining samples constitute the prediction set, with a ratio of 2:1 for the calibration set to prediction set. Finally, 75 samples were allocated to the calibration set and 36 samples were assigned to the prediction set for ABTS, FRAP, and DPPH. The statistical results of the total sample sets and calibration and prediction sets are presented in Table 1. It was evident that for the three antioxidant activity parameters, the mean and standard deviation (SD) values in the calibration sets were close to those of the prediction sets, indicating that the sample division was reasonable and contributed to a stable calibration model.

### 3.3. PLS Models Based on Different Spectral Pretreatment Methods

The raw NIR spectra are heavily overlapped and susceptible to disturbances caused by baseline drift, noise, signal background, light scattering, and sample particle inhomogeneity. Therefore, it is necessary to employ spectral pre-processing methods to improve the robustness and predictive performance of the model. In this study, four different spectral pre-processing methods are discussed and compared, including 1D+SG, smoothing, MSC, and SNV, and the results are presented in Table 2. It was observed that the NIR spectra processed by the SNV method produced better performance for ABTS, FRAP, and DPPH. Additionally, the SNV method yielded high *r*^2^*_m_* values for all three components, and *cR*^2^*_P_* > 0.5 for all three components in the y-randomization test, indicating the absence of random adjustments or overfitting. Therefore, the quantitative models would be constructed based on the SNV processed spectral data.

### 3.4. PLS Models Based on Different Wavelength Selection Methods

#### 3.4.1. Results of Full-PLS Models

The Full-PLS model, often used as the benchmark for multivariate calibration methods, is based on a model constructed from the full spectrum (1000–2500 nm). In PLS modeling, selecting an appropriate number of latent variables (LVs) can mitigate the effects of collinearity, band overlap, and redundant noise on the model. Herein, the optimal number of LVs was determined using a 10-fold cross-validation method by selecting the one with the minimum *RMSECV* value. The results are listed in Table 3. For ABTS, FRAP, and DPPH, the optimal number of LVs for Full-PLS models were found to be 15, 16, and 15, respectively. As shown in Table 3, the Full-PLS models performed well for FRAP, with *R*^2^*_C_* = 0.888, *R*^2^*_P_* = 0.819, *RMSEC* = 2.24 μmol/L, and *RMSEP* = 2.23 μmol/L. However, for ABTS and DPPH, the prediction performance of the Full-PLS model was inferior, with *R*^2^*_C_* = 0.836, *R*^2^*_P_* = 0.649, *RMSEC* = 0.37%, and *RMSEP* = 0.57% for ABTS and *R*^2^*_C_* = 0.831, *R*^2^*_P_* = 0.596, *RMSEC* = 1.83%, and *RMSEP* = 1.91% for DPPH. Considering that including a large number of irrelevant variables from the full spectrum would significantly degrade the NIR model performance, wavelength selection algorithms were utilized to improve predictive performance in subsequent analysis.

#### 3.4.2. Results of GA-PLS Models

**Figure 3:**
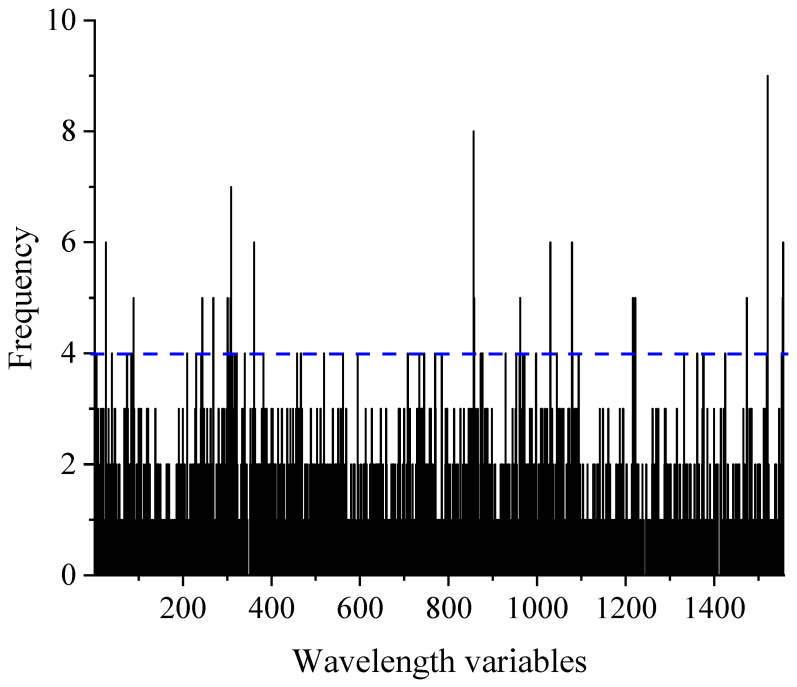
Histogram of selection for each wavelength after 100 runs by genetic algorithm for ABTS. The blue dashed line indicates the boundary.

In order to guarantee the reliability of the GA-PLS model, the number of iterations of the GA procedure was set to 100, while keeping the rest of the parameter settings at their default values in the MATLAB toolbox GA-PLS. Additionally, due to the stochastic nature of the GA, the method was implemented five times for each antioxidant activity parameter and we selected the model with the intermediate *RMSECV* value as the best one. The frequency distribution of wavelength selection from 100 runs of the GA method is shown in Figure 3 (taking ABTS as an example). In this figure, the x-axis represents the number of wavelength variables and the y-axis indicates the selection frequencies of each wavelength. A higher frequency implied a greater likelihood of being selected. As depicted in Figure 3, wavelengths with frequencies greater than or equal to four were considered for selection (indicated by the blue dashed line). Finally, the GA selected 64, 80, and 75 wavelength variables to construct the PLS regression models for ABTS, FRAP, and DPPH, respectively. The results obtained from the GA-PLS models are presented in Table 3, which shows that the optimal LVs for ABTS, FRAP, and DPPH were determined as 15, 14, and 18, respectively, using the GA-PLS model. Comparison between the Full-PLS models and GA-PLS models demonstrated the high accuracy achieved by the GA method. For ABTS, the *Slope* value in the prediction of the independent test set increased from 0.68 to 0.70, and *RMSECV* reduced from 0.61% to 0.53%, indicating the superior performance of GA-PLS compared with the Full-PLS model. For FRAP, the GA showed higher efficiency, with the *Slope* value in the prediction set increasing from 0.77 to 0.81, and *RMSECV* decreased from 3.58 μmol/L to 3.36 μmol/L. As for DPPH, the GA significantly improved the prediction performance of the model. The *Slope* value in the prediction set increased from 0.66 in the Full-PLS model to 0.78 in the GA-PLS model, while *RMSECV* reduced from 3.11% in the Full-PLS model to 2.58% in the GA-PLS model.

#### 3.4.3. Results of CARS-PLS Models

**Figure 4:**
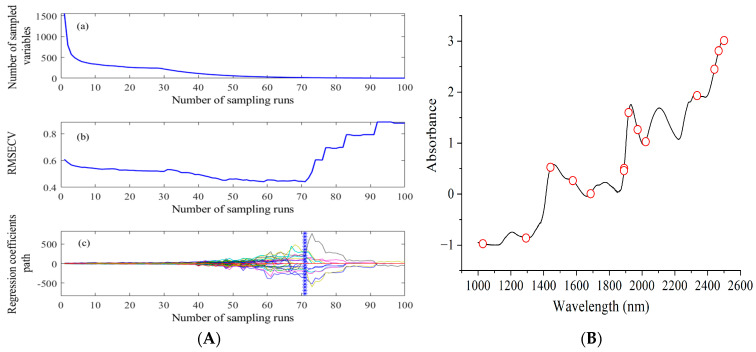

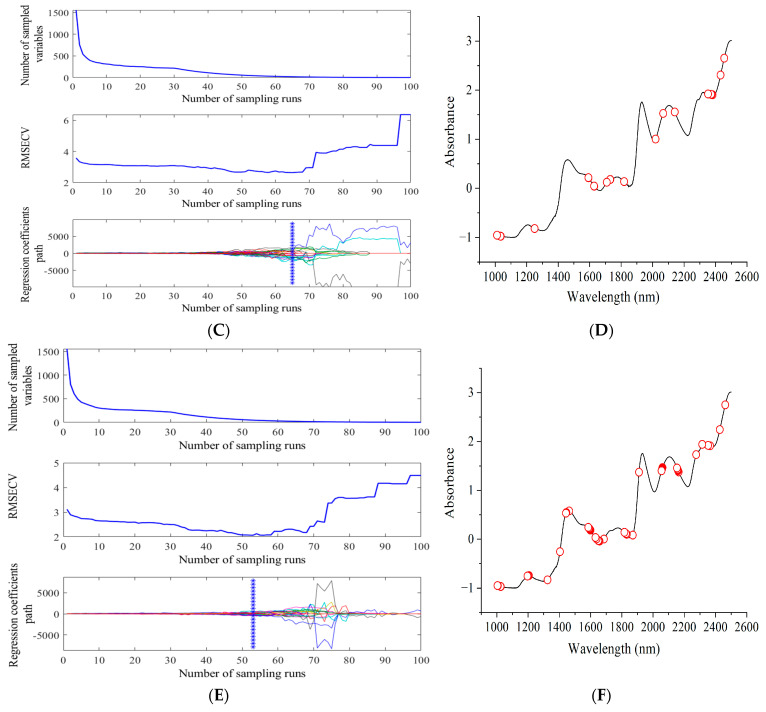
Plots of CARS wavelength selection on spectra for ABTS (**A**), FRAP (**C**), and DPPH (**E**). Plots (**a**–**c**) show the changing trend in the number of sampled wavelengths, *RMSECV* values, and the regression coefficient path of each wavelength with increase in sampling runs, respectively. Each line with different color is composed of the regression coefficient values of each wavelength under all sampling runs. Plots of wavelength distribution by CARS for ABTS (**B**), FRAP (**D**), and DPPH (**F**).

CARS is a multivariate optimization method that is suitable for wavelength selection of high-dimensional data, and can simplify the modeling speed and improve the model accuracy. During the execution of the CARS algorithm procedure, the Monte Carlo sampling was set to 100 times, and 80% of all samples were randomly selected to establish a PLS model in each iteration. The wavelength selection and distribution of CARS for ABTS, FRAP, and DPPH are plotted in Figure 4. Taking ABTS as an example, the variation trend in the number of sampled wavelengths and *RMSECV* values and the regression coefficient path of each wavelength with the number of sampling runs are displayed in Figure 4A(a–c), respectively. In Figure 4A(a), it can be observed that the number of sampled wavelengths decreased sharply as the number of sampling runs increased from 0 to 10, but then showed a gentle trend in the range of 10–100 sampling runs. This is because EDF was employed to remove a large number of irrelevant wavelength variables and ARS was used with the remaining wavelengths with large absolute regression coefficients. As shown in Figure 4A(b), when the number of sampling runs was 71, the *RMSECV* achieved lowest value, indicating that the optimal subset was obtained at the 71st sampling time (marked by the blue asterisk in Figure 4A(c)). However, after 71 sampling runs, the *RMSECV* value showed a fast increase because the key wavelengths were removed, which showed the importance of key wavelengths in calibration model prediction performance. Similarly, for FRAP and DPPH, the minimum *RMSECV* values were obtained when the sampling times were 65 and 52, respectively (Figure 4C,E). Finally, CARS selected 14, 21, and 47 wavelength variables for ABTS, FRAP, and DPPH, respectively. Figure 4B,D,F show the distribution of the wavelengths selected by CARS for ABTS, FRAP, and DPPH. These selected wavelength variables are represented by circles displayed on the spectrum of the optimal spectral pre-processing method. The optimal variables were applied to construct the CARS-PLS model and the results are listed in Table 3. Compared with the GA-PLS models, the CARS-PLS models exhibited better prediction performance in the assessment of the three antioxidant activity parameters. Among the three antioxidant activity parameters, superior performance (the lowest *RMSECV*) was found in the CARS-PLS models used for the prediction of ABTS, FRAP, and DPPH. Overall, the CARS-PLS models performed better than the Full-PLS and GA-PLS models in the prediction of levels of ABTS, FRAP, and DPPH, which have *RMSECV* values of 0.44%, 2.64 μmol/L, and 2.06%, respectively.

### 3.5. Discussion of Results

**Figure 5:**
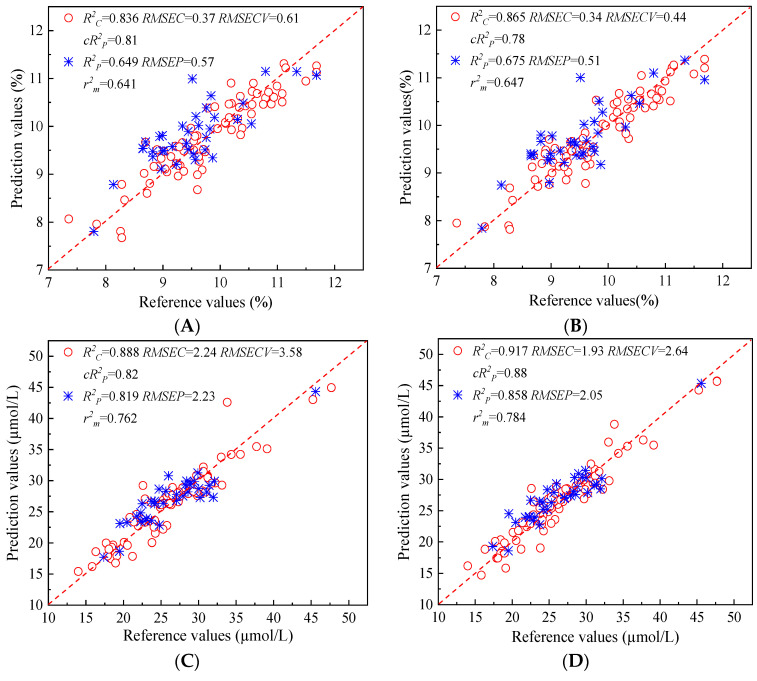

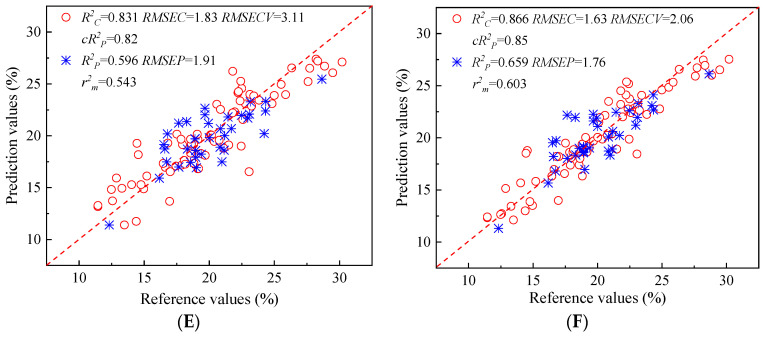
Scatter plots of reference values and prediction values for ABTS (**A**,**B**), FRAP (**C**,**D**), and DPPH (**E**,**F**) and using Full-PLS models (**A**,**C**,**E**) and CARS-PLS models (**B**,**D**,**F**).

The prediction performance of each antioxidant activity parameter decreased in the order CARS-PLS > GA-PLS > Full-PLS, indicating that the CARS wavelength selection algorithm yielded the best result for predicting ABTS, FRAP, and DPPH. This superiority can be attributed to CARS’s ability to avoid overfitting risk through EDF and ARS. By utilizing EDF and ARS, wavelengths with small absolute values of regression coefficients were eliminated, while those with larger absolute values were selected [49]. Moreover, CARS only selected 14, 21, and 47 key wavelengths from the full spectrum (1557) to construct PLS models for ABTS, FRAP, and DPPH, respectively. This significantly reduced the number of selected wavelengths and simplified the model complexity. However, the GA method carries a risk of overfitting which may degrade the model prediction performance. Table 3 presents the results of PLS models based on different wavelength selection algorithms. As for the CARS-PLS models and Full-PLS models, the *RMSEP* for ABTS decreased from 0.57% to 0.51%, for FRAP it was reduced from 2.23 μmol/L to 2.05 μmol/L, and for DPPH it decreased from 1.91% to 1.76%. Additionally, the *RMSECV* values reduced from 0.61% to 0.44% for ABTS, from 3.58 μmol/L to 2.64 μmol/L for FRAP, and from 3.11% to 2.06% for DPPH. Also, there were improvements in *r*^2^*_m_* values for ABTS from 0.641 to 0.647, for FRAP from 0.762 to 0.784, and for DPPH from 0.543 to 0.603, indicating better congruence between measured and predicted values. In the y-randomization test, all *cR*^2^*_P_* values in the CARS-PLS models exceeded 0.5, suggesting no overfitting or random adjustments. In order to further clearly show the superior performance of the CARS-PLS models, Figure 5 illustrates the correlation between reference values and predicted values for ABTS (Figure 5A,B), FRAP (Figure 5C,D), and DPPH (Figure 5E,F) using the Full-PLS models (Figure 5A,C,E) and CARS-PLS models (Figure 5B,D,F). The calibration samples are shown in blue, while the prediction samples are in red. The closer the samples are to the regression line (indicated by the red dashed line), the better the prediction of the model. The results demonstrated that, for either the calibration or validation sets, the predicted values of the CARS-PLS model exhibited better fit with reference values compared to those of the Full-PLS model. This indicates that the CARS-PLS models developed in this study could be effectively utilized for predicting antioxidant activity in *D. officinale*.

## 4. Conclusions

This research confirmed the feasibility of NIR spectroscopy combined with chemometrics methods to predict the antioxidant activity (ABTS, FRAP, and DPPH) of *D. officinale*. Firstly, the PLS models were established using SNV spectral pre-processing methods. Then, different wavelength selection methods were developed to screen key wavelength variables, and their prediction accuracies were compared. The results showed that the CARS-PLS model outperformed other PLS models and yielded the optimal predictions of three antioxidant activity indicators in *D. officinale*. Compared with the Full-PLS models, the *RMSEP* of the CARS-PLS model for ABTS, FRAP, and DPPH decreased by 10.53%, 8.07%, and 7.85%, respectively. The results showed that the established CARS-PLS models were able to effectively improve prediction performance. Overall, this study demonstrated the potential of NIR spectroscopy combined with the CARS-PLS model in the rapid evaluation of the antioxidant activity of *D. officinale*.

## Figures and Tables

**Table 1:** Mean, standard deviation, and range of attributes measured in *Dendrobium officinale* using the reference methods.

Component	Mean	Standard Deviation	Range
Total sample sets			
ABTS (N = 111) (%)	9.7	0.9	7.4–11.7
FRAP (N = 111) (μmol/L)	26.5	6.2	14.0–47.7
DPPH (N = 111) (%)	20.0	4.1	11.4–30.2
Calibration sets			
ABTS (N = 75) (%)	9.8	0.9	7.4–11.7
FRAP (N = 75) (μmol/L)	26.6	6.7	14.0–47.7
DPPH (N = 75) (%)	20.0	4.5	11.4–30.2
Prediction sets			
ABTS (N = 36) (%)	9.5	0.8	7.8–11.7
FRAP (N = 36) (μmol/L)	26.4	5.1	17.4–45.6
DPPH (N = 36) (%)	20.0	3.0	12.3–28.6

**Table 2:** Comparison of results obtained by different spectral pretreatment methods on ABTS, FRAP, and DPPH.

Component	Models	Calibration Sets				y-Randomization		Prediction Sets			
		*R* ^2^ *_C_*	*RMSEC*	*RMSECV*	*Slope*	*R* ^2^ *_rand_*	*cR* ^2^ *_P_*	*R* ^2^ *_P_*	*RMSEP*	*Slope*	*r* ^2^ *_m_*
ABTS (%)	Raw	0.806	0.40	0.62	0.81	0.024	0.79	0.602	0.61	0.64	0.592
	1D+SG	0.749	0.46	0.61	0.75	0.049	0.72	0.487	0.65	0.49	0.478
	MSC	0.837	0.37	0.62	0.84	0.050	0.81	0.587	0.62	0.68	0.532
	SNV	0.836	0.37	0.61	0.84	0.042	0.81	0.649	0.57	0.68	0.641
	Smoothing	0.801	0.41	0.63	0.80	0.017	0.79	0.600	0.61	0.64	0.587
FRAP (μmol/L)	Raw	0.855	2.55	3.73	0.86	0.101	0.80	0.765	2.58	0.81	0.746
	1D+SG	0.974	1.07	3.42	0.98	0.126	0.91	0.751	2.62	0.82	0.712
	MSC	0.888	2.24	3.58	0.89	0.004	0.89	0.819	2.23	0.77	0.762
	SNV	0.888	2.24	3.58	0.89	0.127	0.82	0.819	2.23	0.77	0.762
	Smoothing	0.851	2.59	3.77	0.85	0.075	0.81	0.760	2.61	0.81	0.742
DPPH (%)	Raw	0.813	1.93	3.35	0.82	0.075	0.77	0.578	2.00	0.69	0.486
	1D+SG	0.833	1.83	3.29	0.83	0.125	0.77	0.656	1.82	0.79	0.544
	MSC	0.814	1.93	3.20	0.81	0.060	0.78	0.616	1.89	0.73	0.527
	SNV	0.831	1.83	3.11	0.83	0.017	0.82	0.596	1.91	0.66	0.543
	Smoothing	0.806	1.97	3.38	0.81	0.100	0.75	0.58	1.99	0.69	0.493

**Table 3:** Comparison of results obtained by different wavelength selection methods on ABTS, FRAP, and DPPH.

Component	Models	LVs	Variables	Calibration Sets				y-Randomization		Prediction Sets			
				*R* ^2^ *_C_*	*RMSEC*	*RMSECV*	*Slope*	*R* ^2^ *_rand_*	*cR* ^2^ *_P_*	*R* ^2^ *_P_*	*RMSEP*	*Slope*	*r* ^2^ *_m_*
ABTS (%)	Full-PLS	15	1557	0.836	0.37	0.61	0.84	0.042	0.81	0.649	0.57	0.68	0.641
	GA-PLS	15	64	0.852	0.35	0.53	0.85	0.214	0.74	0.711	0.49	0.70	0.688
	CARS-PLS	14	14	0.865	0.34	0.44	0.86	0.164	0.78	0.675	0.51	0.73	0.647
FRAP (μmol/L)	Full-PLS	16	1557	0.888	2.24	3.58	0.89	0.127	0.82	0.819	2.23	0.77	0.762
	GA-PLS	14	80	0.872	2.39	3.36	0.87	0.058	0.84	0.824	2.31	0.81	0.800
	CARS-PLS	16	21	0.917	1.93	2.64	0.92	0.082	0.88	0.858	2.05	0.80	0.784
DPPH (%)	Full-PLS	15	1557	0.831	1.83	3.11	0.83	0.017	0.82	0.596	1.91	0.66	0.543
	GA-PLS	18	75	0.866	1.51	2.58	0.87	0.019	0.86	0.571	2.11	0.78	0.418
	CARS-PLS	14	47	0.866	1.63	2.06	0.87	0.038	0.85	0.659	1.76	0.73	0.603

## Data Availability

The original contributions presented in the study are included in the article, further inquiries can be directed to the corresponding author.
